# Modulation of host cell signaling during cytomegalovirus latency and reactivation

**DOI:** 10.1186/s12985-021-01674-1

**Published:** 2021-10-18

**Authors:** Nicholas A. Smith, Gary C. Chan, Christine M. O’Connor

**Affiliations:** 1 Department of Microbiology & Immunology, SUNY Upstate Medical University, Syracuse, NY 13210 USA; 2 Department of Genomic Medicine, Infection Biology Program, Lerner Research Institute, Cleveland Clinic, Cleveland, OH 44195 USA

**Keywords:** Cytomegalovirus, HCMV, Latency, Reactivation, Monocytes, CD34^+^ HPCs, Cell signaling

## Abstract

**Background:**

Human cytomegalovirus (HCMV) resides latently in cells of the myeloid compartment, including CD34^+^ hematopoietic progenitor cells and circulating monocytes. Healthy hosts maintain the virus latently, and this infection is, for the most part, asymptomatic. However, given the proper external cues, HCMV reactivates from latency, at which point the virus disseminates, causing disease. The viral and cellular factors dictating the balance between these phases of infection are incompletely understood, though a large body of literature support a role for viral-mediated manipulation of host cell signaling.

**Main body:**

To establish and maintain latency, HCMV has evolved various means by which it usurps host cell factors to alter the cellular environment to its own advantage, including altering host cell signaling cascades. As early as virus entry into myeloid cells, HCMV usurps cellular signaling to change the cellular milieu, and this regulation includes upregulation, as well as downregulation, of different signaling cascades. Indeed, given proper reactivation cues, this signaling is again altered to allow for transactivation of viral lytic genes.

**Conclusions:**

HCMV modulation of host cell signaling is not binary, and many of the cellular pathways altered are finely regulated, wherein the slightest modification imparts profound changes to the cellular milieu. It is also evident that viral-mediated cell signaling differs not only between these phases of infection, but also is myeloid cell type specific. Nonetheless, understanding the exact pathways and the means by which HCMV mediates them will undoubtedly provide novel targets for therapeutic intervention.

## Background

**Fig. 1:**
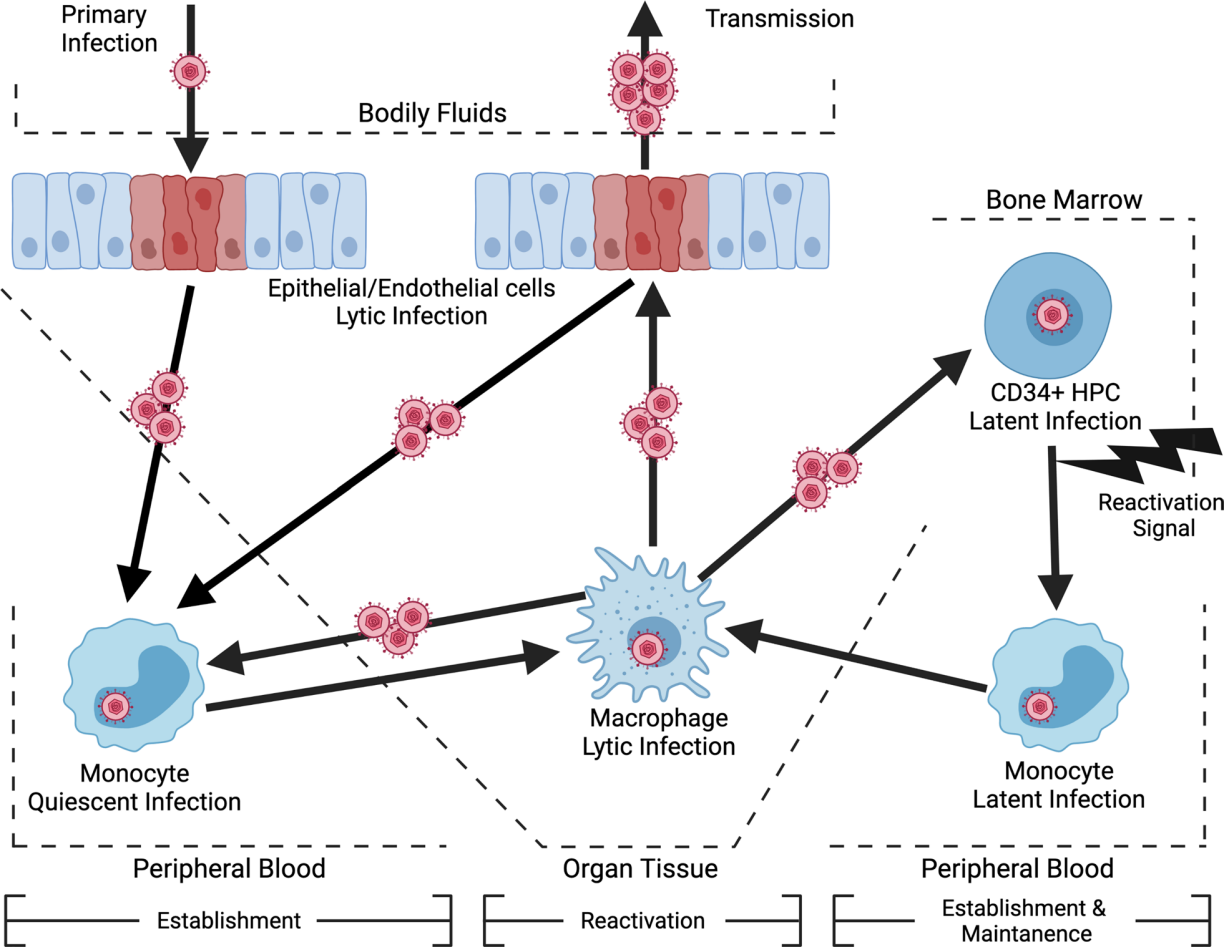
The myeloid compartment and HCMV latency. During a primary infection, HCMV is spread through bodily fluids to oral epithelial cells, which are permissive for lytic infection. HCMV then infects peripheral blood monocytes, resulting in a unique form of latency known as a quiescent infection. Cellular signaling induced by viral entry drives the survival, extravasation, and monocyte-to-macrophage differentiation of infected monocytes. Viral spread to peripheral organs leads to lytic infection of tissue endothelial and epithelial cells. Additionally, infected monocytes can travel to the bone marrow and spread HCMV to CD34^+^ HPCs, which are the long-term latency reservoir. Following an external reactivation signal, latently infected HPCs preferentially differentiate down the myeloid lineage into latently infected monocytes, and ultimately into replication permissive macrophages. Overall, the myeloid compartment is essential to viral life cycle and allows for the life-long persistence of HCMV

HCMV latency is maintained in cells of the myeloid compartment, specifically peripheral blood monocytes and CD34^+^ hematopoietic progenitor cells (HPCs) [1–6]. During the initial stages of a primary infection, HCMV lytically infects and amplifies within epithelial cells, ultimately leading to infection of peripheral blood monocytes [7, 8]. HCMV infection of monocytes results in a unique form of latency, which has been termed a quiescent infection by the Yurochko Lab [9–13]. The establishment of this quiescent infection is characterized by the lack of viral lytic replication and limited expression of latency-associated viral gene products [3, 4, 14]. However, the maintenance phase of this distinct form of latency is limited, as the viral entry process triggers signaling events that extend monocyte survival beyond their normal 48-h lifespan, enhance migration, and stimulate differentiation into replication permissive macrophages [9, 10, 15–19], which together allows monocytes to serve as vehicles of viral dissemination to peripheral tissue. Once infected monocytes extravasate into tissue and differentiate into macrophages, viral replication and spread can occur [14, 20, 21]. Importantly, spread to and infection of the bone marrow leads to the establishment of a latent reservoir within CD34^+^ HPCs [2, 5, 22]. In contrast to a quiescent infection of monocytes during a primary infection, long-term maintenance occurs in latently infected CD34^+^ HPCs and requires an external activation stimulus for reactivation into lytic replication [23]. In contrast, the early signaling events following infection of monocytes drives their differentiation into macrophages and spontaneous viral reactivation at 2–3 weeks post-infection. [13], an important aspect distinguishing a quiescent infection from more canonical definitions of latency in CD34^+^ HPCs. Following reactivation signal(s), latently infected CD34^+^ HPCs preferentially differentiate down the myeloid lineage into latently infected monocytes and ultimately replication permissive macrophages, leading to the reseeding of virus in peripheral organs sites [24, 25]. Similarly, quiescently infected monocytes can also be stimulated to reactivate prior to 2–3 weeks with an external reactivation signal (e.g. ref. [26]). Thus, the early HCMV-induced events contributing to the establishment, maintenance, and reactivation of quiescently infected monocytes and latently infected CD34^+^ HPCs are likely very similar. Thus, studies on quiescently infected monocytes will also likely provide insight into the mechanisms of latency in CD34 + HPCs and vice versa. The establishment of a persistent infection in both monocytes and CD34^+^ HPCs is critical for viral dissemination and life-long persistence within an infected host (Fig. 1). Herein, we review the host cell signaling pathways HCMV coopts to make the cellular environment more amenable to latency establishment, as well as maintenance and reactivation.

## Main text

### Establishment

#### Establishment of latency

**Fig. 2:**
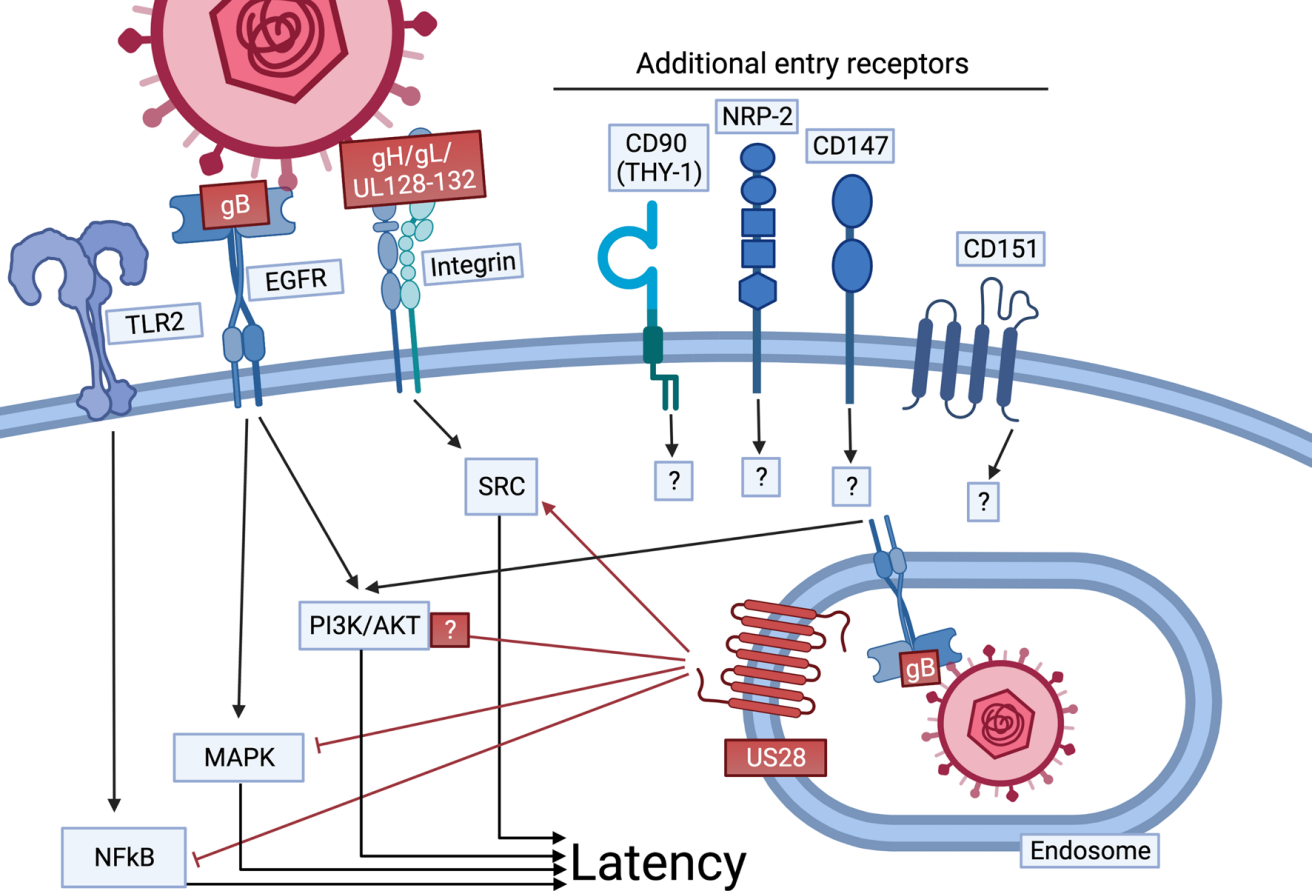
Regulation of receptor signaling induced by viral glycoproteins is required for HCMV latency establishment. During HCMV entry in myeloid cells, glycoprotein complexes engage cellular receptors, including EGFR, integrins, and TLR2, stimulating a complex network of signaling pathways that generates an environment necessary for the establishment of HCMV latency within the myeloid compartment. However, many of the same signaling pathways also promote MIEP activity, and thus must be “fine-tuned” to ensure the MIEP remains inactive. Evidence indicates that viral factors carried by the incoming virion, such as US28, actively regulate virus-mediated cellular pathways to promote the establishment of latency

The establishment of latent infection requires the restriction of viral gene expression from the major immediate early promoter (MIEP) to abrogate HCMV lytic replication. The MIEP controls the expression of viral immediate early (IE) genes, which are responsible for initiation of the viral lytic replication program [27, 28]. Restriction of the MIEP is accomplished by the repression of activating transcription factors, binding of repressive transcription factors, and by the chromatin rearrangement leading to inaccessibility of the promoter (reviewed in [29]). Although literature is limited on the mechanisms specifically attributed to the establishment of latency, several studies hint at the modulation of cellular signaling pathways during the viral entry process as essential. This section will focus on how cellular receptors and viral G protein-coupled receptors (vGPCRs) modulate cellular signaling pathways during the viral entry process in order to promote the establishment of HCMV latency (Fig. 2).

#### HCMV entry and signaling in myeloid cells

Entry of HCMV is a complicated process that allows for efficient entry of the virus but also initiates signaling events that alter the cellular environment. In the absence of viral gene expression during the establishment of latency, these signaling events are posited to be critical for latency establishment. Initially, the HCMV glycoproteins gM/gN bind heparin sulfate proteoglycans in a reversible, low-affinity interaction [30], which is rapidly replaced with irreversible high affinity binding between viral glycoproteins to cellular receptors (reviewed in [31]). The viral glycoprotein gH is found in three complexes within the virion; the dimeric gH/gL, the trimeric gH/gL/gO, or the pentameric gH/gL/UL128-131 complex [32–34]. For the infection of monocytes, the pentameric complex is required [35, 36]. gH directly binds integrin β1, while UL128-131 binds β3 integrins [19, 37]. Additionally, the viral glycoprotein gB binds epidermal growth factor receptor (EGFR) on both monocytes and CD34^+^ HPCs [19, 38–41]. EGFR is a major determinant of HCMV tropism for the myeloid compartment, as monocytes are the only human leukocyte that expresses EGFR, which is also expressed in myeloid cell lines used for HCMV latency models, such as THP-1 cells [39, 42, 43]. EGFR is an important receptor for efficient infection and establishment of latency in CD34^+^ HPCs [40]. Additional cellular receptors for HCMV have been identified, including platelet derived growth factor receptor alpha (PDGFR-α), neuropilin-2 (NRP-2), thymocyte differentiation antigen-1 (THY-1), olfactory receptor family 14I1 (OR14I1), CD147, and CD151 [44–48]. Many of these receptors, such as PDGFR-α and OR14I1, are not expressed on cells of the myeloid lineage, and thus their involvement in the establishment of latency is unlikely [39, 48–51]. Despite the known expression of receptors such as NRP-2, THY-1, CD147, and CD151 on myeloid cells, the contribution of these HCMV entry receptors to infection of monocytes or CD34^+^ HPCs have yet to be thoroughly examined [52–55].

In addition to the triggering of signaling pathways by viral glycoprotein interactions with cellular receptors, HCMV encodes four vGPCRs, including US28, US27, UL33, and UL78 that can modulate cellular signaling pathways. While all four vGPCRs are de novo synthesized during lytic infection, only US28, UL33, and UL78 are expressed during latency (reviewed in [56]). Each of the HCMV-encoded GPCRs is incorporated into the mature viral particle [57–61], thereby facilitating their delivery into the host cell upon viral fusion. Importantly, virion-delivered US28 is sufficient to attenuate early IE gene expression [57, 62]. However, HCMV fails to maintain long-term latency in the absence of de novo synthesized US28, indicating virion delivered US28 is sufficient for the establishment, but not maintenance, of HCMV latency [62]. While UL78 and UL33 are also incorporated into the virion, the role for these vGPCRs during the establishment of latency in myeloid cells has yet to be explored, although UL33 functions during reactivation, as detailed below [63]. Consequently, we will focus on the potential role of US28 in regulating glycoprotein induced cellular signaling pathways to promote the establishment of HCMV latency.

#### PI3K/Akt

EGFR is a well-studied viral entry receptor that contributes to the establishment of efficient infection and latency of both monocytes and CD34^+^ HPCs [19, 38, 41, 64]. During HCMV entry, viral glycoprotein gB binds EGFR, triggering the activation of downstream PI3K/Akt signaling [19, 38–40]. HCMV activation of EGFR induces a non-canonical PI3K/Akt signaling pathway, via activation of Sh2 domain containing inositol 5-phospatase 1 (SHIP1) [16, 19], which results in the preferential phosphorylation of Akt at serine 473. In contrast, canonical growth factor induced PI3K/Akt signaling within monocytes stimulates the dual phosphorylation of Akt at both serine 473 and threonine 308 [16]. Akt activated by HCMV infection leads to the upregulation of a unique subset of anti-apoptotic proteins, including myeloid cell leukemia-1 (Mcl-1) protein, heat shock protein 27 (HSP27), and X-linked inhibitor of apoptosis (XIAP), necessary for the survival of infected monocytes [9, 15, 17, 18]. Although the upregulation of these survival factors is critical for allowing the establishment of latency within naturally short-lived monocytes, how the non-canonical EGFR/PI3K/Akt signaling induced by HCMV directly contributes to the repression of MIEP is not entirely clear. In CD34^+^ HPCs, Kim et al. showed inhibition of EGFR following genome nuclear translocation increases IE gene expression while attenuating expression of the latency maintenance protein, UL138 [40]. Chronic EGFR and PI3K signaling is also necessary to maintain latency as inhibition of the pathway stimulates viral reactivation, suggesting EGFR/PI3K/Akt signaling may directly regulate MIEP activity through modulation of activating and repressive transcription factors. In support, the EGFR/PI3K/Akt cascade directly regulates the activities of several transcription factors. Alternatively, early EGFR induced signaling events regulate viral genome trafficking within endosomes in both monocytes and CD34^+^ HPCs [40, 65]. The viral tegument protein pp71 mediates the removal of promyelocytic leukemia nuclear body (PML-NB) proteins, including Daxx and histone deacetylases (HDACs), from the MIEP to allow transcription initiation [66–68]. However, Lee and Kalejta showed pp71 is sequestered to endosomes in TB40/E-latently infected CD34^+^ HPCs, thereby preventing pp71 translocation to the nucleus [69], possibly rendering pp71 unable to degrade PML-NB proteins during latency establishment. Similarly, Saffert and Kalejta showed pp71 is restricted from the nucleus in AD169-infected N-Tera2 or THP-1 cells [67], two in vitro model cell types to study HCMV latency. Using these same cell systems and virus strain, HDAC inhibitor treatment or siRNA-mediated knockdown of Daxx resulted in IE gene expression [67]. However, subsequent reports from the Sinclair [70] and Stamminger [71] groups, who employed embryonal carcinoma NT2D1 cells or THP-1 monocytic cells, respectively, revealed a different phenotype. Daxx knockdown in NT2D1 cells did not impact IE gene transcription following infection with the Toledo strain [70], and similarly, knockdown of PML, Daxx, or Sp100 failed to initiate IE gene expression in TB40/E-infected THP-1 cells [71]. These data suggest the underlying mechanisms indeed may prove distinct, based on viral strain, clinical versus lab-adapted strain, and/or cell type used in the study. Nonetheless, it is intriguing to speculate that the EGFR driven PI3K/Akt control of endosomal trafficking may contribute to latency establishment by sequestration of pp71 or other viral proteins. Regardless of the mechanism of action, these data suggest a direct role for the EGFR/PI3K/Akt pathway in the early suppression of the MIEP and the establishment of latency. Although GCPRs are known alter the PI3K/Akt cascade, the role of viral GCPRs in modulating glycoprotein-induced EGFR/PI3K/Akt signaling to promote latency establishment within HCMV-infected monocytes and CD34^+^ HPCs remains to be elucidated and an important avenue of research.

#### MAPK

Mitogen activated protein kinases (MAPKs), including c-Jun N-terminal kinases (JNK) 1/2/3, extracellular signal-regulated protein kinases (ERK) 1/2, and p38, promote transcription from the MIEP. MAPK signaling pathways activate the Activator Protein-1 (AP-1) transcription factor, comprised of c-fos and c-jun, that bind to the MIEP to initiate transcription [72]. ERK1/2 signaling mediates cyclic AMP response element binding protein (CREB)-dependent activation of the MIEP [73]. In addition to activating transcription factors, derepression of the MIEP by inhibition of transcriptional repressors plays an equally important role during reactivation and IE gene expression. The MIEP associates with heterochromatin protein 1 (HP-1), a chromosomal protein implicated in gene silencing, in latently infected monocytes and CD34^+^ HPCs [74–76]. However, the MIEP and HP1 association is lost during HCMV reactivation. Mechanistically, Dupont et al. demonstrated that ERK stimulates the activities of mitogen and stress-activated kinases 1 and 2 (MSKs), which recognize and subsequently phosphorylate CREB to promote transcription and phosphorylation at serine 10 of histone H3, resulting in the de-stabilization of histones with HP1 during IL-6-mediated reactivation within dendritic cells (DCs) [77]. Finally, the MIEP and subsequent viral replication are also activated in a p38-dependent manner [78], further revealing the importance of MAPK activity to MIEP activation. Despite the essential role of MAPKs in promoting IE gene expression, which we discuss in more detail below, MAPKs are rapidly activated by viral entry into monocytes and CD34^+^ HPCs without initiating transcription from the MIEP. HCMV gB triggers ERK/MAPK to promote the expression of Mcl-1 and the subsequent survival of infected monocytes and CD34^+^ HPCs [79]. Additionally, and as detailed below, EGFR signals through MEK/ERK to activate the early growth response 1 (EGR-1) transcription factor, which drives the expression of the viral latency maintenance protein UL138 [80]. Rapid secretion of IL-1β from HCMV-infected monocytes triggers p38 MAPK signaling that promotes a cellular environment conducive for latency [81]. These studies highlight the critical importance of MAPK signaling in promoting a cellular environment supportive of latency, despite also functioning in stimulating IE gene expression. Thus, the question remains as to how the activation of MAPKs are able to promote the establishment of latency. As discussed in more detail below, US28 attenuates ERK phosphorylation when expressed in isolation in THP-1 cells [82]. Accordingly, US28 reduces the expression and phosphorylation of c-fos [62]. Similarly, infection with a US28 deficient virus increased AP-1 binding to the MIE enhancer/promoter [62] and IE gene expression in monocytes [62, 82]. Interestingly, c-jun is also downregulated in latently infected CD34^+^ HPCs and Kasumi-3 cells following HCMV infection, albeit in a US28-independent manner [62, 83]. However, it remains unclear if virion delivered US28 plays a role in regulating glycoprotein-activated MAPKs. It is intriguing to hypothesize that MAPKs are activated upon entry through glycoprotein/receptor interactions, which is then subsequently countered by US28. However, it is important to note that MAPKs are not completely attenuated by US28; rather US28 acts as a rheostat that fine-tunes the activity of this signaling pathway. Therefore, there may be a threshold level of activation that is important for the establishment of latency, but is not sufficient to initiate MIEP-driven transcription.

#### Src

Integrins are a family of heterodimeric receptors composed of a single α and β chain. There are 24 α and 9 β integrin chains that can form 25 individual receptors expressed to different levels depending on cell type (reviewed in [84]). Each combination of α and β chain not only has distinct binding properties, but also exhibits different downstream signaling characteristics. HCMV utilizes the integrin diversity to mediate entry into different cell types and to initiate distinct cell-type specific signaling. During entry into fibroblasts, HCMV engages only the α_2_β_1_, α_6_β_1_ [85], or αvβ_3_ [86] integrin heterodimer via the gH/gL/gO trimer, which stimulates transient Src signaling. In contrast, the pentameric complex simultaneously binds both β_1_ and β_3_ integrin containing heterodimers to stimulate a chronic but low-level activation of Src [87]. Importantly, the trimeric complex has no effect on Src activity during entry into monocytes, suggesting that Src-mediated signaling specifically initiated from the pentameric complex is critical to the establishment of infection [37]. During lytic infection of fibroblasts, there is also evidence that gB binds β1 integrins through a disintegrin domain binding [88]. However, the contribution of this interaction to the induction of Src signaling remains unexplored. Importantly, we recently reported gB directly interacts EGFR, but not with β1 integrins, in infected monocytes [19]. In monocytes, pentamer-induced Src signaling is required for increased cellular motility as well as proper endocytosis and trafficking of the virion [37, 41, 87, 89]. However, whether Src signaling regulates the MIEP in monocytes or CD34^+^ HPCs is unclear. Recently, Src family kinases (SFKs) were implicated in the regulation of chromatin structure at the MIEP. As described in detail below, the upregulation of SFKs, Src and hematopoietic cell kinase (Hck), during latency recruit the monocytic leukemia zinc finger protein (MOZ) histone acetyltransferase leading to chromatin rearrangement and initiation of transcription from the MIEP [77]. These data suggest that glycoprotein-mediated activation of Src signaling during viral entry must be restricted to a certain extent to allow for the cellular changes necessary for the establishment of latency while also suppressing the MIEP. In support of this, expression of US28 in THP-1 cells, a model myeloid cell line, results in the downregulation of Src gene expression [62]. It is important to point out that Krishna et al. showed THP-1 cells transduced with a constitutively expressed US28 construct increase Src phosphorylation in a phosphokinase study, though these data were not subsequently confirmed in this study [82]. However, supporting this work, Aslam et al. showed that Src phosphorylation was upregulated in the presence of US28 [90]. It is unclear from this work, however, which Src phospho-site was evaluated, which is critical, as Ser^416^ phosphorylation renders Src active, while phosphorylation at Ser^527^ is a negative regulatory site, associated with Src inactivity (reviewed in [91]). Whether virion-delivered US28 represses early Src signaling to promote the establishment of latency remains to be elucidated, though this is an attractive hypothesis.

#### NF-κB pathway

Nuclear factor-kappa B (NF-κB) signaling is crucial for many aspects of HCMV biology [92], but the role it has in the establishment of latency is unclear. Adding to the complexity of the regulation of this pathway, HCMV encodes both agonists and antagonists of NF-κB. As a transcription factor, NF-κB binds to the MIEP to drive IE gene expression [93, 94], and during lytic infection, virus binding and entry activates NF-κB signaling and expression of the MIEP [34, 95]. Interestingly, upon monocyte infection, NF-κB is robustly activated by viral glycoprotein and cellular receptor interactions in a Toll-like receptor-2 (TLR-2) dependent manner to promote the induction of a distinct inflammatory phenotype in monocytes [39, 96, 97]. The NF-κB-mediated phenotype stimulates the expression of an unusual milieu of inflammatory and anti-inflammatory cytokines and chemokines likely important for driving extravasation of HCMV-infected monocytes in tissue. However, the question still remains as to why activation of the NF-κB pathway during infection of undifferentiated myeloid cells does not lead to the expression of IE genes, as it does during lytic infection. One possibility is that NF-κB-driven cellular gene expression is functional, but other regulated cofactors not active in undifferentiated myeloid cells are required to stimulate IE expression. Krishna and colleagues have demonstrated US28 regulates NF-κB nuclear localization during latency. A US28 signaling deficient mutant increased nuclear localization of NF-κB, suggesting US28 attenuates the NF-κB signaling pathway by an unknown mechanism [82]. Additionally, functional viral microRNAs (miRNAs), including those known to regulate NF-κB [92], are delivered to the host cell by the infecting virion [98]. These data suggest a potential model whereby HCMV stimulates NF-κB activity via glycoprotein-cellular receptor interactions, but limits its activity through US28 and miRNAs, in order to allow for the expression of NF-κB responsive cellular genes without initiation of transcription from the MIEP.

### Maintenance and reactivation

**Fig. 3:**
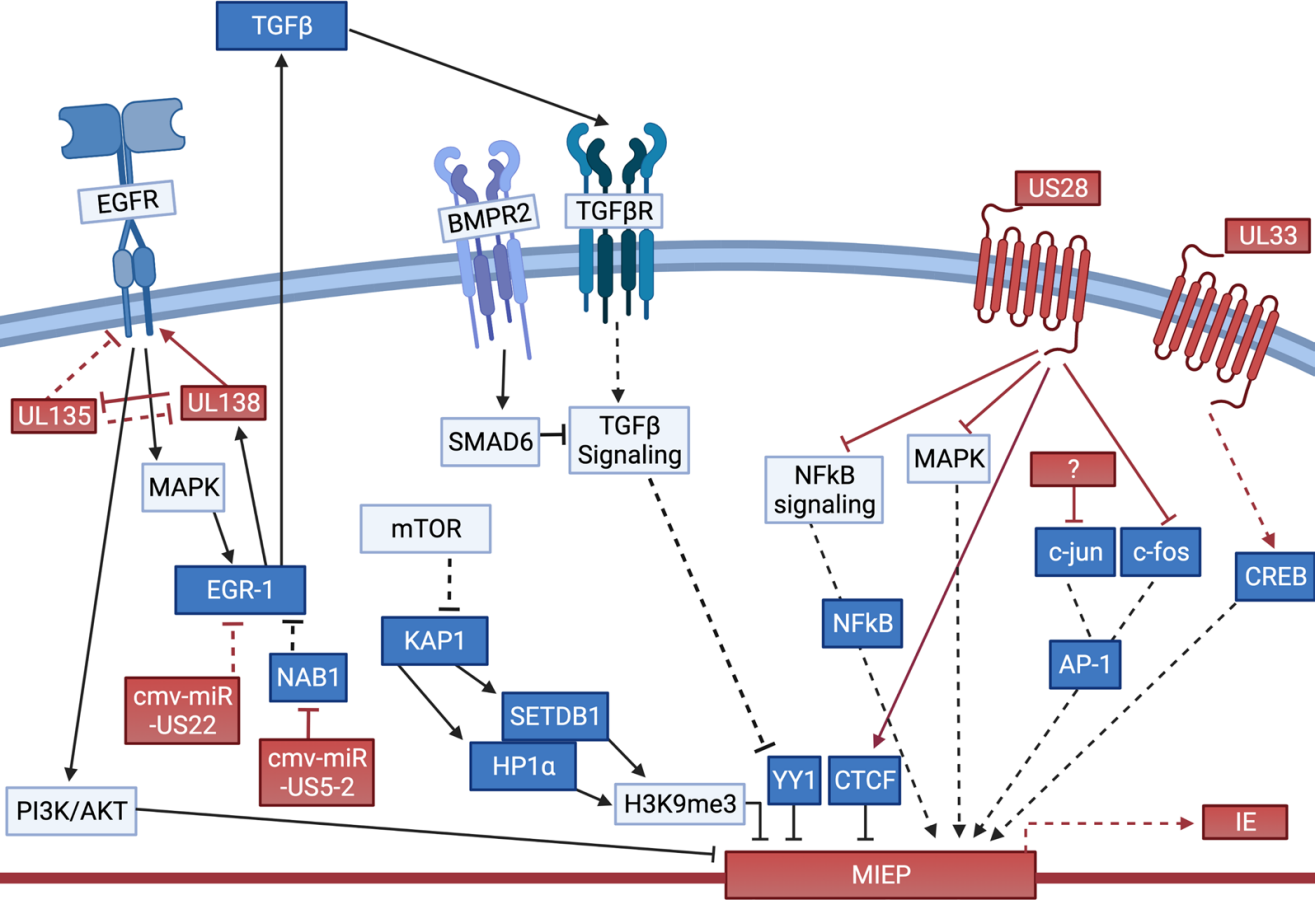
Modulation of signaling pathways during latency maintenance. Multiple signaling pathways are modulated by viral factors to support latency maintenance. Cellular receptors like EGFR, BMPR2, and TGFβR are usurped by HCMV to mediate downstream cellular pathways, including MAPK and TGFβ. HCMV also encodes its own factors, such as the viral GPCR, US28, which regulates several cellular signaling cascades. Additionally, these host and viral receptors modulate downstream transcription factor activity. The cumulative effect of this collective regulation is to alter the cellular environment to support latency maintenance and prevent expression from the MIE enhancer/promoter. Conversely, when provided the proper external cues, HCMV again alters host cell signaling, thereby making the cell more amenable to viral reactivation. Cellular factors are shown in blue, and viral factors are shown in red. Pathways activated during latency are shown in solid lines, and processes activated during reactivation and suppressed during latency are shown in dotted lines

Once the virus establishes latency, the virus must now devise ways to maintain this phase of infection. This process is undoubtedly multifaceted, but it is clear HCMV has co-evolved with its host, usurping host cell networks to its own benefit. An “easy target” for the virus is cellular signaling, as this is one of the prime means to alter the cellular milieu. Indeed, HCMV has devised biological mechanisms to coopt host cell signaling to maintain viral latency and trigger reactivation into the lytic cycle (Fig. 3).

#### The UL133-138 locus

Work from a variety of groups have revealed that the UL133-138 locus is quite important to the regulation of latency and reactivation (reviewed in [99]). UL138 is required for the establishment of latency, and in fact, abrogation of this open reading frame (ORF) results in an infection that favors lytic replication, leading to increased virion production [100–102]. More recently, Buehler et al. showed UL138 regulates EGFR signaling, which downstream, upregulates PI3K/Akt signaling [80, 103]. Pharmacological inhibition of PI3K/Akt favors reactivation, though this phenotype is most significant when combined with reactivation stimulating cytokines, suggesting other factors regulate this pathway [80]. These data also highlight that viral manipulation of host cell signaling is likely not as simple as a direct on/off state, but instead reflects a mechanism that is more fine-tuned. In fact, UL138 actually regulates its own expression during HPC latent infection, which it accomplishes through the upregulation of the EGFR-regulated transcription factor, EGR-1. This host protein binds the viral genome, thereby driving *UL138* transcription in HPCs, as well as fibroblasts [80], in turn creating a feedback loop regulating UL138-mediated events. Supporting the role of this feedback loop towards latency, disruption of the EGR-1 binding site upstream of UL138 in the viral genome results in the inability for the virus to establish/maintain latency in HPCs [80].

The importance of the UL133-138 locus does not end with viral latency. While UL138 helps maintain latency, UL135 is critical for efficient reactivation. When given the proper reactivation cues, UL135 counters UL138-mediated functions by targeting EGFR [80]. Indeed, disruption of the UL135 ORF results in a virus that fails to efficiently reactivate in CD34^+^ HPCs [104]. To ensure EGFR signaling and its downstream effectors are appropriately regulated, HCMV additionally encodes an miRNA, cmv-miR-US22, which targets EGR1 [105]. UL135’s role during reactivation does not end with countering UL138’s functions. UL135 interacts with host adapter proteins, Abelson-interactor (Abi)-1 and Cbl-interaction protein (CIN) 85/ CD2 associated protein (CD2AP), which in turn regulate EGFR on the cell surface. Hence, in the absence of UL135 and its interactions with these host proteins, EGFR is increased on the cell surface of HPCs, thereby amplifying signaling and favoring latency. In line with this, inhibiting EGFR or its downstream pathways leads to reactivation when coupled with reactivation stimuli and rescues the reactivation defect observed for UL135 mutant viral infections [80, 103]. Collectively, both UL135 and cmv-miR-US22 antagonize UL138-mediated EGFR regulation, thereby creating a cellular environment more amenable to reactivation. These data also illuminate the critical nature of EGFR signaling during latency, for which the virus has devised several means to regulate this pathway.

#### Manipulation of host cell signaling impacts MIE enhancer/promoter activity

A key step in establishing and maintaining HCMV latent infection is silencing of the MIE locus, which is likely initiated by chromatin remodeling (reviewed in [29]). The MIE enhancer/promoter is thought of as the “lytic switch”, acting like what may more accurately be described as a rheostat to skew the infection towards one that is latent versus one that is lytic (reviewed in [29]). Long regarded as a silencing factor of the MIE enhancer/promoter [106, 107], Poole and colleagues recently confirmed the requirement for yin yang 1 (YY1) transcription factor binding for maintaining latency [108]. Perhaps unsurprisingly, HCMV regulates YY1 by manipulating host cell signaling. Host-encoded transmembrane serine/threonine kinase, bone morphogenetic protein receptor 2 (BMPR2) signaling induces SMAD6, a SMAD family member that negatively regulates BMP and transforming growth factor β (TGFβ) signaling (reviewed in [109]). In the context of latent infection, SMAD6 upregulation restricts the activity of TGFβ receptor (TGFβR) [108]. This is critical, as latently infected induced pluripotent stem cells (iPSCs) or CD34^+^ HPCs display a significant upregulation of TGFβ [108, 110, 111], mediated at least in part by cmv-miR-US5-2 attenuation of NGFI-A binding protein 1 (NAB1) [112]. Since NAB1 is a transcriptional repressor of EGR-1 [113, 114], this represents an additional mechanism by which HCMV ensures EGR-1 transcription and subsequent TGFβ production. Additionally, cmv-miR-UL22A also targets TGFβ signaling, and in fact deletion of the pre-miR-UL22A sequence within the viral genome results in a viral mutant that is less efficient at reactivation [110]. This is consistent with the finding that increased TGFβ signaling leads to an increase in the host-encoded miRNA, hsa-miR-29, which ultimately targets YY1. In turn, recruitment of YY1 to the MIE enhancer/promoter is decreased, which relieves the repression of the viral promoter and leads to reactivation [108]. Collectively, these findings reveal not only the importance of TGFβ signaling to latency and reactivation, but the critical nature of this pathway towards regulating a central transcription factor that contributes to the balance between the active and repressive states of the MIE enhancer/promoter. This latter point is amplified by the multiple biological mechanisms HCMV has devised to regulate cell signaling that culminates at YY1 regulation.

A region rich in transcription factor binding sites, the MIE enhancer/promoter locus is studded with multiple binding sites for those which activate this very strong promoter region. Thus, just as the virus must evolve strategies to only recruit silencing transcription factors like YY1 during latency, HCMV has converse tactics to recruit transcription factors that activate the MIE enhancer/promoter as the virus reactivates. Investigators have shown that several of these transcription factors are regulated by viral manipulation of host cell signaling. For example, Keller an colleagues found transcription from the MIE locus was derepressed in quiescently infected NTera2-derived neuronal cells treated with forskolin, a compound that phosphorylates CREB [115, 116]. Indeed, this was reliant upon the CRE-binding sites located within the MIE distal enhancer [115]. More recently, Kew et al. showed phosphorylated CREB binding to the MIE enhancer/promoter aids in viral reactivation in DCs, which is dependent upon the activation of the ERK-MSK signaling axis. Consistent with this, deletion of the CREB binding sites in the MIE enhancer/promoter region results in a mutant virus unable to reactivate in DCs, though both CD14^+^ monocytes and immature DCs maintained viral genomes. It is also important to point out that in this context, CREB not only acts as a canonical transcription factor, but it also promotes the phosphorylation of histone H3, which aids in chromatin remodeling of the MIEP, facilitating reactivation [73]. More recently, we showed a parallel mechanism for regulating CREB activity and recruitment to the MIE enhance/promoter. Consistent with previous findings in other cell types (e.g. COS-7, fibroblasts; [117, 118]), we found signaling via the viral GPCR, UL33, activates CREB [63]. Furthermore, UL33-mediated signaling facilitates recruitment of phospho-CREB to the MIE locus during reactivation. Indeed, disruption of the entire UL33 ORF or UL33’s G-protein coupling motif (the ‘DRY’ motif) results in a failure to reactivate from latency following infection of CD34^+^ HPCs, despite the ability of each mutant virus to maintain viral genomes. While phospho-CREB was recruited to the MIE locus in latently infected Kasumi-3 hematopoietic cells treated with 12-O-tetradecanoylphorbol-13-acetate (TPA) to induce reactivation [63, 119], this was significantly reduced in parallel cultures infected with either UL33 mutant [63]. Since cellular GPCRs coupled to Gα_o_ activate CREB via p38 MAPK [120], it is plausible UL33 uses a similar mechanism. However, inhibition of p38 MAPK in monocytes had little impact on phospho-CREB binding to the MIE locus [73]. Thus, additional work is needed to comprehensively understand the upstream mechanisms underlying CREB regulation.

NF-κB and AP-1 host transcription factors also function to activate the MIE enhancer/promoter (reviewed in [29]). Latently infected Kasumi-3 hematopoietic cells stimulated with tumor necrosis factor (TNF) α to induce reactivation [119] and treated simultaneously with curaxins to inhibit NF-κB, results in a significant decrease in *UL123* transcription, when compared to cultures treated with TNFα alone [121]. Furthermore, the HCMV-encoded GPCR, US28 attenuates NF-κB during latent infection [82], consistent with the requirement of US28 expression and signaling for viral latency, discussed in detail below [25, 57, 62, 82, 122–126]. In fact, pharmacological inhibition of NF-κB in monocytes infected with a US28-deletion viral mutant resulted in an infection that favored latency rather than the lytic-like phenotype infection with this mutant usually observed [82]. With four binding sites within the MIE enhancer/promoter (reviewed in [29]), it is likely NF-κB’s role during reactivation is key. Further work elucidating the exact mechanisms by which US28, for example, modulates host signaling to regulate this important transcription factor is warranted and may reveal hematopoietic-specific signaling cascades critical for viral reactivation.

AP-1 is a heterodimeric transcription factor, comprised of c-fos and c-jun subunits [127]. We have shown previously that both c-fos [62] and c-jun [128] are attenuated during latency, thereby limiting their heterodimerization. The balance of AP-1 binding to the MIE enhancer/promoter is key to its regulation; while the absence of AP-1 binding aids in keeping the MIE enhancer/promoter silenced [62], its binding to the promoter proximal site is required for viral reactivation [129]. Despite a requirement of this transcription factor for reactivation, however, AP-1 binding is dispensable for lytic replication in fibroblast or epithelial cells [72, 129]. The upstream signaling events regulating fos and jun are currently under investigation, and while we have shown US28-induced signaling targets fos [62], the viral and/or cellular factors manipulating jun are unknown. As discussed in more detail below, the signaling cascade US28 hijacks to attenuate c-fos remains to be elucidated, but it is likely that the virus balances the activation and attenuation of signaling cascades to skew the cellular milieu towards one that favors latency versus one that aids in reactivation. Thus, viral proteins, like US28, are likely antagonized to “switch” their functions during reactivation, similar to the relationship between UL138 and UL135.

A recent study detailed the involvement of Kruppel-associated box domain-associated protein (KAP)-1/ tripartite motif-containing (TRIM) 28 and mammalian target of rapamycin (mTOR) during latency and reactivation in CD34^+^ HPCs [130]. KAP-1 co-regulates transcription, as it recruits SET domain bifurcated (SETDB) 1 and HP1α, which facilitate H3K9me3. This histone modification is a repressive chromatin mark, and during HCMV latency, represses the MIE locus after SETDB1 and HP1α recruitment (reviewed in [29]). As a result, these factors silence the MIE locus throughout latency. However, when mTOR is activated, it phosphorylates KAP-1, relieving chromatinization of the MIE locus, leading to activation of lytic gene transcription and the production of viral particles, suggesting a role for this pathway in both latency and reactivation [130]. Supporting this, and as mentioned above, work from Buehler et al. reveal treatment with either an Akt or PI3K pharmacological inhibitor stimulates lytic replication in CD34^+^ HPCs cultured under latent conditions [80]. Additionally, we have shown HCMV stimulates mTOR activity 24 h post-latent infection of monocytes [17, 18], though this activity was not sufficient to drive active replication [18]. This could reflect differences in cell type specificity or cell environment at distinctive times during latent infection (e.g. early [24hpi] vs. later [7dpi] events). Alternatively, this supports the notion that mTOR is regulated in a rheostat-like fashion, where a threshold of activation has to be met or has not been reached. While the mechanism(s) through which this pathway is regulated remain unknown, rapamycin, an mTOR inhibitor, administered to transplant recipients suppresses viral reactivation [131–134]. Whether this is due to a direct impact on the virus or the immune response is debated [135], since rapamycin failed to impact *UL123* expression in LPS-stimulated DCs [136]. MAPK and Akt signaling axes regulate downstream mTOR signaling, all of which are implicated in entry and maintenance of CMV in cells supporting latency [137]. Akt is activated rapidly following latent infection of monocytes [16, 18, 138] and CD34^+^ HPCs [80], though it is attenuated by 72hpi in monocytes [16, 138] and minimally sustained in CD34^+^ HPCs [80]. Similarly, mTOR signaling is also rapidly upregulated within 24hpi of monocytes [17, 18], which is attenuated during latency maintenance [130]. Additionally, sustained pharmacological inhibition of Akt activity results in reactivation of WT, latent virus in CD34^+^ HPCs [80], suggesting completely abrogating Akt activity for prolonged times alters the cellular environment, such that it no longer is amenable to supporting HCMV latency.

#### Manipulation of MAPK signaling

The importance of MAPK signaling to HCMV latency and reactivation has become increasingly clear over the past decade. Several studies have shown MAPK signaling promotes HCMV reactivation in monocyte-derived DCs [73, 77, 139]. However, this regulation is not binary; like Akt, low-levels of MEK and ERK phosphorylation are maintained during HCMV latent infection [80], arguing activity of these MAPK proteins is fine-tuned. Additionally, such subtle differences may reflect tissue- or cell type-specificity. In support of this, MAPK activation promotes reactivation in a cell type specific manner [139, 140], suggesting that not all cells that harbor CMV latently do so similarly (reviewed in [141]). Indeed, IL-6 mediated activation of MAPK signaling facilitates viral reactivation in monocyte-derived-DCs and monocyte-derived-Langerhans-like cells (LCs), although viral reactivation was not coupled with activation of IL-6-mediated MAPK signaling in LCs [77, 140], suggesting involvement of other viral or host factors. As mentioned above, SFKs, specifically Src and Hck, play important roles in this cell type-specific signaling (reviewed in [141]). Both of these SFKs display upregulated expression during reactivation in monocyte-derived-DCs, and while MAPK activity impacts histone phosphorylation at the MIEP, chromatinization is regulated in parallel in an SFK-dependent fashion via the recruitment of MOZ histone acetyltransferase (HAT) [77]. Upstream of SFK signaling are various receptors capable of potentiating signals, one of which is the receptor tyrosine kinase, Fms related receptor tyrosine kinase 3 receptor (FLT-3R), which downstream, regulates a cascades such as Ras and ERK/MAPK (reviewed in [142]). Crawford et al. recently identified pUL7 as a novel, secreted ligand for the FLT-3R using HEK293T cells. This ligand-receptor interaction indeed leads to cellular signaling, and in bone marrow lymphoblast cells, PI3K/Akt and MAPK signaling cascades are activated. In turn, pUL7 induces differentiation of both HPCs and monocytes. Supporting these data, the investigators found that pUL7 required for reactivation [143].Thus, pUL7 activation of MAPK signaling may represent another mechanism HCMV has devised to ensure viral reactivation. Whether Src and/or Hck aid in regulating the pUL7-FLT-3R cascade to impact downstream MAPK signaling remains unknown, but it is attractive to hypothesize these factors coordinate their functions to ensure proper MAPK activity during viral reactivation. Furthermore, such regulation may in fact be cell type dependent, underscoring the need to interrogate such pathways across hematopoietic cell model systems. To this point, the addition of MEK or ERK inhibitors in combination with reactivation stimuli significantly increases viral reactivation compared to reactivated CD34^+^ HPCs in the absence of the inhibitors [80]. These data reveal: 1) MEK/ERK inhibition alone is not sufficient to drive reactivation, 2) significant increases in MEK/ERK phosphorylation tip the balance towards reactivation, and 3) MAPK activity as it pertains to HCMV latency and reactivation may depend on cell type. Collectively, these data reveal MAPK signaling is a key pathway usurped by HCMV during latency and reactivation, and like Akt, is likely fine-tuned by the virus to skew the host cell environment to favor a specific phase of infection.

#### US28-mediated regulation of viral latency and reactivation

US28 has long been considered a latency-associated transcript, as early as 1998 when Patterson and colleagues showed this viral GPCR was detected in the peripheral blood mononuclear cells of infected individuals [144]. Shortly thereafter, Beisser et al. were the first to demonstrate *US28* was transcribed during latent infection of THP-1 monocytic cells [145]. Until recently, however, a role for US28 during this phase of infection had not been described. We were the first to demonstrate the requirement for US28 during viral latency [57], a finding subsequently confirmed independently by several groups [25, 82, 122–124, 126]. US28 is a potent signaling molecule [146], thus it is unsurprising that US28-mediated signaling helps establish and maintain viral latency [57, 62, 82, 147]. Incorporation of US28 into the mature viral particle [57, 62] allows for its immediate expression, facilitating silencing of the MIE promoter/enhancer as early as 2 days post-infection of hematopoietic cells [62]. To this end, US28 attenuates MAPK and NF-κB signaling [82], as well as fos expression downstream [62]. That US28 regulates the MAPK pathway is consistent with previous studies showing the upregulation of MAPK signaling promotes HCMV reactivation in monocyte-derived DCs [73, 77, 139], detailed above. US28-mediated attenuation of MAPK signaling is also consistent with downstream suppression of fos, which ultimately prevents the AP-1 transcription factor from binding and activating the MIE promoter/enhancer [62]. As detailed above, preventing recruitment of AP-1 to this promoter region is critical for successful latent infection, and conversely, its binding is essential for viral reactivation [129]. As an active signaling protein expressed during latency, it is perhaps not surprising that US28 regulates host factors that ultimately impact the activity of the MIE enhancer/promoter, since this region is so crucial to balancing latency and reactivation. Recently, Elder et al. demonstrated US28 also regulates CCCTC-binding factor (CTCF) binding to the MIE enhancer/promoter. CTCF binding to this region increases during latency, thereby suppressing transcription from the MIE locus. Indeed, this is dependent upon US28-mediated signaling [122]. Furthermore, these new data reveal that the neutrophil chemoattractants, S100A8 and S100A9, which are downregulated during latency [148], are in fact regulated, at least in part, by US28-mediated recruitment of CTCF to their promoter [122], revealing yet another means by which US28 manipulates cellular proteins, rendering the host cell more amenable to viral latency.

As much as we have learned collectively as a field, there are many outstanding questions surrounding US28’s function(s) during latency that remain. For the signaling pathways US28 regulates that are identified to-date, it is clear from that US28 alters the cellular milieu during latency in a ligand- [57, 62, 82, 126, 147] and G protein-coupling-dependent fashion [57, 62, 82, 126]. US28 binds a variety of cellular chemokines and couples to various G proteins (reviewed in [56]), thus understanding the key, cellular proteins US28 usurps to its advantage will inform potential treatment strategies. Latently infected granulocyte macrophage progenitors (GMPs) display an increase in the expression of the CC chemokine, monocyte chemotactic protein-1 (MCP-1) [149], a known US28 ligand (reviewed in [56]). Pharmacological inhibition of G_α_ proteins with pertussis toxin or PI3K with wortmannin attenuated MCP-1 transcript levels [149], suggesting this is regulated via GPCR-mediated signaling. Though the molecular mechanism(s) underpinning MCP-1’s regulation during latency is yet to be elucidated, it is certainly plausible the viral-encoded GPCRs expressed during latency [57, 63, 145, 150, 151], such as US28, may leverage MCP-1’s upregulation to its own advantage, perhaps promoting dissemination in the initial stages of viral infection.

While the numerous host cell chemokines are obvious potential ligands for US28, it is equally plausible US28 interacts with a viral-encoded chemokine. HCMV encodes viral chemokines and cytokines (reviewed in [152]), thus, this could represent a novel mechanism by which US28 regulates host cell signaling in hematopoietic cells, thereby retaining the virus in its latent state until given the proper cues. While US28 is not required for viral reactivation in hematopoietic cells [25, 82, 122–124, 126], Crawford et al. published expression of the complete, functional US28 ORF has no impact on maintaining latency, but is required for viral reactivation in CD34^+^ progenitor cells isolated from fetal liver. [147]. This difference is possibly explained by tissue origin of the cells (fetal liver-derived vs. hematopoietic-derived). Nonetheless, since this viral GPCR is expressed throughout all stages of infection, some host or viral factor(s) likely influence US28 during reactivation in hematopoietic cells to either overcome its strong “pro-latent” signaling, or “switch” its signaling to favor lytic infection.

#### miRNA regulation of latency and reactivation

Both host cell- and viral-encoded miRNAs have functions during latency and reactivation (reviewed in [153]). The known functions for cmv-US5-2, cmv-miR-UL22A, and cmv-miR-US22 are discussed above. Several other CMV-encoded miRNAs also regulate cell signaling pathways during latency and reactivation. For example, cmv-miR-US25-1 targets RhoA, and disruption of this viral-encoded miRNA increases the proliferation of CD34^+^ HPCs [154]. As part of the Rho family of GTPases, RhoA acts as a switch for a variety of signaling cascades as it cycles between its inactive GDP-bound and active GTP-bound states (reviewed in [155]). How RhoA might be manipulated and coopted by viral signaling proteins and other factors during latency in hematopoietic cells is unclear, though Diggins et al. hypothesize a role for TGFβ signaling [154], which regulates the RhoA pathway [156–161]. If true, this would reveal yet another means by which HCMV attenuates the TGFβ cascade during latency. As discussed above, cmv-miR-UL22A targets SMAD3 to prevent robust TGFβ signaling during latent infection of CD34^+^ HPCs [110]. Thus, it is possible that cmv-miR-US25-1 and cmv-miR-UL22a function cooperatively to ensure this host cell signaling pathways is dampened during latent infection. Similarly, cmv-miR-UL148D, which is robustly expressed during viral latency, targets the activin signaling axis in monocytes, by directly suppressing the activin A receptor type (ACVR) 1B cellular receptor, which in turn limits the secretion of IL-6 [162]. This represents a possible mechanism by which the virus subverts immune detection. Additionally, cmv-miR-UL148 targets the cellular immediate early response gene 5 (IER5). Repression of IER5 results in an increase in host-encoded cell division cycle 25B (CDC25B) expression, which aids in suppressing *UL123* transcription while simultaneously increasing cyclin-dependent kinase 1 (CDK1). Thus, cmv-miR-UL148-mediated regulation of the IER5-CDC25B axis is important for latent infection of Kasumi-3 and primary CD34^+^ cells [163]. cmv-miR-US5-1 and cmv-miR-UL112 also function to alter host cell signaling pathways during latency. Hancock and colleagues recently found these two viral-encoded miRNAs downregulate host cell Forkhead box O3a (FOXO3a). While both miRNAs protect CD34^+^ HPCs from apoptosis [164], whether their expression and targeting of FOXO3a is required for latency remains outstanding. FOXO3a binds and drives transcription from the MIE internal promoter 2 (iP2) [165], a promoter that aids in reactivation of latent virus in primary CD34^+^ HPCs [166] and Kasumi-3 cells [129]. Furthermore, mutation of the FOXO binding sites within the MIE promoter/enhancer locus leads to inefficient viral reactivation following stimulation of latently infected CD34^+^ HPCs [165]. Thus, it seems plausible that cmv-miR-US5-1 and cmv-miR-UL112 target FOXO3a to limit sufficient quantities of this protein, such that it cannot transactivate the MIE locus. cmv-miR-UL112 may indeed have dual functions in this regard, as Lau et al. showed this miRNA targets IE72 in both monocytes and THP-1 monocytic cells to aid in the maintenance of latency [167], consistent with earlier work [168]. HCMV also modulates host cell miRNAs to suppress the MIE-encoded proteins. Indeed, hsa-miR-200 family members target IE86. Mutation of the seed sequence in the *UL122* 3’ untranslated region (UTR) results in a virus that fails to undergo latency in Kasumi-3 cells. Expression of this family of host-encoded miRNAs are upregulated in cells that favor latency (e.g. Kasumi-3, CD34^+^, and monocyte cells) [169], thus it is attractive to speculate that they may also target proteins involved in signal transduction networks. Collectively, both viral and host miRNAs are pivotal to latency and reactivation. Many of the changes these non-coding RNAs impart are small, yet significant. This again highlights that the regulation of factors involved in cellular signaling are indeed fine-tuned.

## Conclusions

It is quite evident that HCMV usurps host cell signaling to its advantage, beginning as early as the initial phases of latency establishment and through reactivation. Arguably, viral-manipulation of these signaling cascades alters the cellular milieu, making it more amenable to viral latency. Indeed, such changes to the cell environment are further altered following external cues that trigger viral reactivation; pathways that were attenuated to establish and maintain latency become activated (and vice versa). However, it is important to realize the regulation of host cell signaling is not binary. Such cell signaling pathways are more likely finely regulated, where the slightest of change in activity results in profound cellular changes. This is particularly evident during the establishment of latency where signaling pathways important for generating the biological changes critical to supporting latency are often also activated during lytic infection to promote viral gene expression and replication. This paradox reveals the intricacy of viral manipulation of host cell signaling during latency establishment and maintenance, as well as reactivation. Cellular signaling cascades are intertwined, and their regulation is most likely dependent on multiple viral and cellular factors working in coordinated fashion. Further work is necessary to unravel the regulatory mechanisms employed by HCMV to “rewire” the complex cellular signaling network that promote establishment, maintenance, and reactivation of HCMV latency within the myeloid compartment. Finally, viral manipulation of host signaling cascades is likely cell type specific depending on the type of infection elicited by HCMV. Quiescent infection of primary monocytes likely produces a signaling network skewed towards promoting the establishment of latency while signaling within latently infected hematopoietic cells is undoubtedly more conducive to both the establishment and long-term maintenance of latency. In turn, cell type specific signaling likely leads to differences in the activation signals necessary for reactivation into lytic replication. Thus, understanding how HCMV modulates cell signaling in the cells that support viral latency and reactivation will undoubtedly provide clues as to the pathways crucial to supporting these exact phases of viral infection, keeping in mind that even the cell type used for experimentation matters (e.g. monocyte versus CD34^+^ HPC). As more work is done in this area, we will likely identify pathways worthy of exploiting as novel therapeutic targets of the latent reservoir.

## Data Availability

Not applicable.

## Abbreviations

HCMV: Human cytomegalovirus
HPCs: Hematopoietic progenitor cells
MIEP: Major immediate early promoter
IE: Immediate early genes
vGPCRs: Viral G protein-coupled receptors
UL: Unique long
EGFR: Epidermal growth factor receptor
PDGFR-α: Platelet derived growth factor receptor alpha
NRP-2: Neuropilin-2
THY-1: Thymocyte differentiation antigen-1
OR14I1: Olfactory receptor family 14I1
US: Unique short
PI3K: Phosphoinositide 3 kinase
SHIP1: Sh2 domain containing inositol 5-phospatase 1
Mcl-1: Myeloid cell leukemia-1
HSP27: Heat shock protein 27
XIAP: X-linked inhibitor of apoptosis
PML-NB: Promyelocytic leukemia nuclear body
Daxx: Death domain associated protein
HDACs: Histone deacetylases
MAPKs: Mitogen activated protein kinases
JNK: C-Jun N-terminal kinases
ERK: Extracellular signal-regulated protein kinases
AP-1: Activator Protein-1
CREB: Cyclic AMP response element binding protein
HP1: Heterochromatin protein 1
MSK: Mitogen and stress-activated kinases 1 and 2
DCs: Dendritic cells
EGR-1: Early growth response 1
IL-1β: Interleukin-1β
SFKs: Src family kinases
Hck: Hematopoietic cell kinase
MOZ: Monocytic leukemia zinc finger protein
NF-κB: Nuclear factor kappa B
TLR-2: Toll-like receptor-2
miRNAs: MicroRNAs
ORF: Open reading frame
Abi: Abelson-interactor 1
CIN85: Cbl-interacting protein of 85 kDa
CD2AP: CD2 associated protein
YY1: Yin yang 1
BMPR2: Bone morphogenic protein receptor 2
BMP: Bone morphogenic protein
TGFβ: Transforming growth factor beta
TGFβR: Transforming growth factor beta receptor
iPSCs: Induced pluripotent stem cells
NAB1: NGFI-A binding protein 1
TNFα: Tumor necrosis factor alpha
TPA: 12-O-tetradecanoylphorbol-13-acetate
KAP-1/TRIM28: [Kruppel-associated box domain]-associated protein-1/tripartite motif-containing 28
mTOR: Mammalian target of rapamycin
SETDB1: SET domain, bifurcated 1
H3K9me3: Histone H3 lysine 9 trimethyl
MEK: Mitogen-activated protein kinase kinase 1; MAPK/ERK kinase 1
LCs: Langerhans-like cells
IL-6: Interleukin-6
HAT: Histone acetyl transferase
FLT3: Fms related receptor tyrosine kinase 3
FLT3R: FLT3 receptor
HEK293T: Human embryonic kidney cells 293 transformed
CTCF: CCCTC-binding factor
GMPs: Granulocyte macrophage progenitors
CC: C–C motif chemokine
MCP-1: Monocyte chemotactic protein-1
ACVR1B: Activin A receptor type 1B
IER5: Immediate early response gene 5
CDC25B: Cell division cycle 25B
CDK1: Cyclin-dependent kinase 1
FOXO3a: Forkhead box O3
